# Evaluation of Root Dilaceration in Permanent Anterior and Canine Teeth in the Southern Subpopulation of Iran Using Cone-Beam Computed Tomography

**DOI:** 10.30476/dentjods.2022.95451.1874

**Published:** 2023-09

**Authors:** Safoora Sahebi, Alireza Razavian, Neshat Maddahi, Bahar Asheghi, Maryam Zangooei Booshehri

**Affiliations:** 1 Dept. of Endodontics, School of Dentistry, Shiraz University of Medical Sciences, Shiraz, Iran; 2 Postgraduate Student, Dept. of Endodontics, Dental School, Shiraz University of Medical Science, Ghasrdasht Street, Shiraz, Iran; 3 Dentist, School of Dentistry, Shiraz University of Medical Science, Shiraz, Iran; 4 Oral and Maxillofacial Radiology, Shiraz University of Medical Sciences, Shiraz, Iran

**Keywords:** Cone-Beam Computed Tomography, Canine teeth, Teeth abnormalities, Panoramic radiography

## Abstract

**Statement of the Problem:**

Developmental anomalies such as dilacerations can affect the eruption pattern of permanent anterior teeth. They are characterized by a curvature in the crown and roots of the teeth relative to their normal axis. This anomaly can cause some complexities in routine dental treatments such as root canal treatment, orthodontics, and surgery.

**Purpose:**

The purpose of this study was to assess the prevalence of dilaceration in maxillary and mandibular anterior and canine teeth in Shiraz, Iran using cone-beam computed tomography (CBCT).

**Materials and Method:**

In this retrospective study, a total of 1537 encompassed 400 CBCT images collected from 4 private radiology clinics in Shiraz were assessed. Each tooth was radiographically examined in order to diagnose root dilacerations considering their location (apical, middle, coronal), position in the jaw (maxillary or mandibular), direction (mesial, distal buccal and palatal/lingual), and severity of dilaceration (mild, moderate, and severe). The obtained data were analyzed by Chi-square statistical test and Fisher’s exact test.

**Results:**

In this study, out of 1537 studied teeth, 5.98% had dilaceration. The maxillary and mandibular canine teeth (9.8% and 9.7%, respectively) were significantly the most common teeth involved in this anomaly (*p*< 0.001). The distal direction with mild severity in the apical third of the root was also the most common result obtained from this study (p<0.001). In addition, there was no statistically significant relationship between gender and type of jaw regarding the prevalence of dilaceration in the studied dental groups (p=0.670 and p=0.231, respectively).

**Conclusion:**

In the current research, it was demonstrated through CBCT records that the prevalence of dilaceration in maxillary and mandibular anterior and canine teeth is relatively uncommon. The most prevalent dilaceration was found to be distal direction with mild severity in the apical third of the root.

## Introduction

Developmental dental anomalies are a group of morphological variations that include deviation of normal size, shape, and structure [ 1 ]. Dilaceration is one of the developmental anomalies that defined as a curvature or deviation with different angles in the crowns and roots relative to the normal axis of the teeth. However, in general, there is no clear and precise definition in the studies [ 1 - 3 ]. For example, Hamasha *et al*. [ 4 ] and Malcic *et al*. [ 5 ] considered dilaceration as the curvature of 90 degrees or more compared with Chohayeb *et al*. that consider a curvature greater than 20 degrees or more as dilacerations [ 6 ]. Also Sanatana *et al*. [ 7 ] provided classifications based on the degree of dilaceration (mild, moderate, and severe). 

The exact and main cause of this abnormal morphology is not completely clear. However, it can be due to the mechanical trauma to the calcified portion of a developing tooth, syndromes, and ectopic development of tooth germs [ 8 ]. In permanent anterior teeth, trauma such as intrusion to the associated primary teeth can displace the radicular parts of tooth germ in permanent teeth [ 9 ]. Consequently, trauma is a possible cause for this condition in the anterior teeth area. Nevertheless, it is not the main cause and is still suspected as a pathogenicity [ 4 , 10 ]. It has been stated that traumas such as intrusion and avulsion to primary teeth are not common before the age of four when the formation of the associated permanent tooth roots has not yet begun. Hence, ectopic development can be a more definite cause for dilacerations [ 4 , 11 - 12 ].

Dilaceration is an anomaly that can challenge diagnosis, treatment, and prognosis. Therefore, it is important to know about its occurrence and presence through radiography and clinical examination before the treatment process [ 5 , 8 ]. Based on conventional radiographs, the prevalence of dilaceration in central maxillary incisors has been reported to be between 0.5% and 3.7% [ 4 - 5 ]. Malcic *et al*. [ 5 ] stated that the roots of incisors are often found in the apical third, whereas in the molars they are in the middle third. Moreover, Park *et al*. [ 13 ] presented that the curvature of the roots of maxillary lateral incisors was in the distal and palatal directions. 

There is no consensus on which jaw and which teeth are more prone to dilacerations. Malcic *et al*. [ 5 ] have reported that maxillary anterior teeth and maxillary premolar teeth have a higher prevalence of this anomaly than their associated mandibular teeth.

Most previous studies have used conventional methods to investigate the prevalence of dilacerations [ 2 , 5 ]. If the root has a mesial or distal curvature, dilaceration can be clearly seen on conventional radiography. However, if the curvature is palatal or labial, it may be seen as a target or bulbous eye and only its presence can be determined in the buccal or lingual directions and its exact severity and location cannot be specified [ 14 - 15 ]. Failure to recognize the multiplanar nature of root canal dilaceration is one of the factors associated with the poor outcome of treatment of lateral maxillary incisors compared with multi-root teeth [ 16 - 17 ]. Due to the advantage of three-dimensional cone beam computed tomography (CBCT) radiographs in eliminating distortion and superimposition, the multiplanar nature of the roots can be better recognized [ 18 - 20 ].

Depending on the severity and location of dilaceration, the treatment and prognosis of teeth with this anomaly can vary. With three-dimensional radiographs, the exact location and severity of dilaceration are determined and the awareness of dentists about its prevalence is increased. However, most studies on the prevalence of dilaceration have been performed based on conventional radiography and CBCT has been used only in case reports [ 10 , 21 ]. 

The aim of this study was to investigate the prevalence of dilaceration in anterior and canine teeth in maxillary and mandibular jaws using CBCT. In addition, the direction, severity, and location of this anomaly in the roots of these teeth as well as the relationship between gender and the prevalence of dilaceration in the patient referred to the radiology clinics of Shiraz, Iran were also studied.

## Materials and Method

In this cross-sectional and retrospective study in the period from 2015 to 2021, high-quality CBCT radiographic images archived in four private radiology clinics in Shiraz were used. 400 CBCT scans were randomly selected. They included 184 men and 216 women (age range: 18 to 65 years). Since the CBCT images were obtained from the patients due to a variety of reasons including implants, jawbone fractures, and the diagnosis and treatment of tumors, the patients were not exposed to unnecessary radiation. The Ethics Committee of Shiraz University of Medical Sciences (IR.SUM-S.REC.1397.201) approved this study.

In this study, 400 CBCT images were evaluated including 1537 anterior teeth and canines that were eligible for the study. Of these, 792 were maxillary anterior and canine teeth and 745 were mandibular anterior and canine teeth.

The teeth selected for this study were fully developed concerning their roots and apices. In addition, supernumerary teeth, teeth with internal and external resorption, primary, or permanent teeth with open apices, cleft palates, and impacted teeth were excluded from the study.

The data were obtained from the number of dilaceration in all the patients and the number of dilaceration from CBCT images, the number of teeth, gender of patients, position of the teeth in the jaw (maxillary or mandibular),and the severity of dilaceration based on Santana’s classification [ 7 ]. Moreover, the direction of dilaceration (mesial, distal, buccal, and palatal/lingual), its location (apical, middle, and coronal), the number of teeth with S-shaped roots, and the tooth type were statistically analyzed.

**Figure 1:**
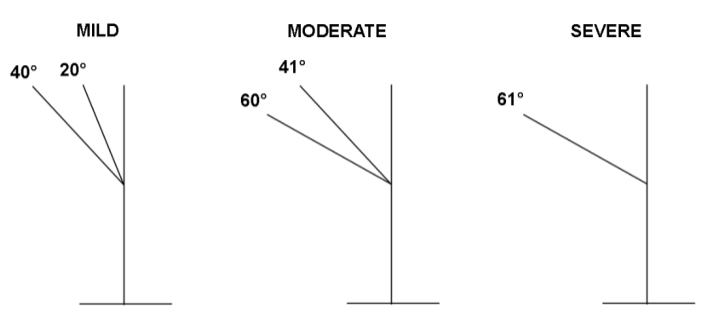
Diagram showing rates - classification of dilaceration in mild, moderate or severe. Source: Santana, Consolaro and Tavano [ 7 ], Schneider [ 22 ]

In this study, the definition of dilaceration and the severity of dilaceration were defined based on the study of Chohayeb *et al*. [ 6 ] and Santana *et al*. [ 7 ], respectively. Chohayeb *et al*. [ 6 ] considered a curvature of greater than 20 degrees from the longitudinal axis of the tooth as dilaceration. In addition, according to the study of Santana *et al*. [ 7 ], the curvature severity was considered mild (20-40°), moderate (41-60°), and severe (more than 60°) (Figure 1).

Moreover, Schneider’s method was used to investigate the curvature angle of dilacerations [ 22 ]. In this method, the angle between two hypothetical lines connecting the apex and the orifice to the beginning of the curvature is measured. In teeth that had multiplanar dilaceration, the largest angle in any direction was considered as dilaceration.

All the CBCT images were obtained by employing a Planmeca Promax 3D Mid device (Helsinki, Finland) at 90 kVp and 14 Ma, with an exposure time of 15 s, and automatically adjusted according to the patients’ body weight and size. A voxel size of 150 μ, a maximum field of view of 10*10 cm, and a high-definition mode were employed.

**Figure 2:**
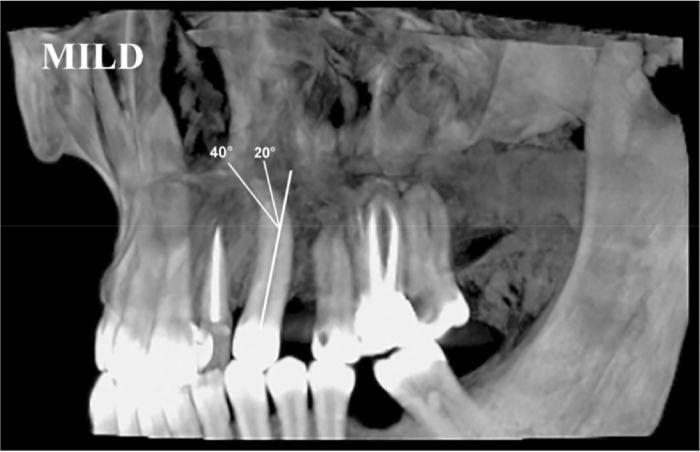
Mild dilaceration (20-40°) in the left maxillary canine in the 3D plane

Root dilaceration was assessed by utilizing a magnification tool in the Romexis software. The CBCT images in the sagittal, coronal, and axial sections were analyzed by the Romexis imaging software (version: 3.8.2) on a 32-inch monitor in dim light (Figure 2).

A senior dental student and a calibrated endodontist examined all the CBCT images twice, retrospectively and independently. There was a two-week interval between the evaluations. Before the experiments, the investigators assessed 60 other CBCT images. If any disparity was observed between the opinions, a radiologist assessed the images to reach a consensus. To evaluate the intra-examiner reliability, a re-assessment was conducted one month after the first session.

The collected data were then analyzed using the SPSS software (SPSS Inc., Chicago IL, USA, version: 18.0) and the figures were generated by employing the GraphPad Prism software (version: 8.0). The Chi-square statistical test and Fisher’s exact test were utilized to assess the qualitative data and the significance level was set at *p*<0.05. In the current study, the *p*Value was utilized to investigate whether root dilaceration was statistically dependent on gender, jaw type, and tooth type. The prevalence of dilacerations and S-shaped roots in the studied teeth was given as percentages. The agreements between the inter- and intra-examiners were computed using Cohen’s kappa coefficient.

## Results

**Figure 3:**
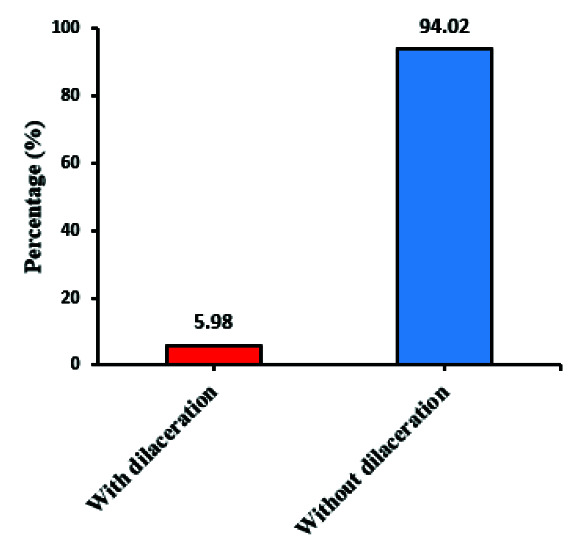
The prevalence of dilaceration in the studied teeth

The prevalence of dilaceration was 14.5% (n=58) among the individuals who had at least one tooth with dilaceration. It was observed that the prevalence of dilaceration in anterior and canine teeth out of 1537 teeth was 5.98% (n=92) (Figure 3). Moreover, the prevalence of S-shaped roots was 0.1% (n=2).

**Figure 4:**
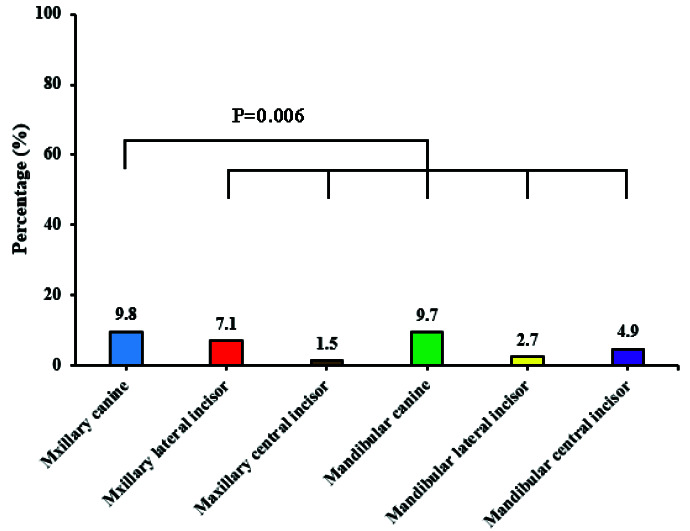
The prevalence of dilaceration in each studied tooth

**Figure 5:**
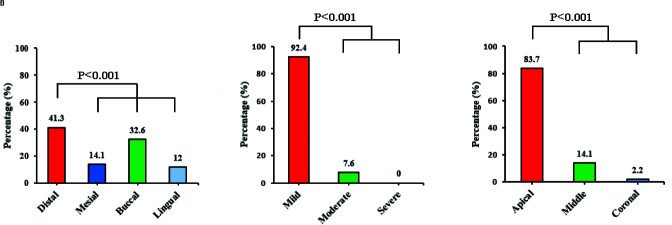
**a:** The direction, **b:** Severity, and **c:** Prevalence of dilaceration in the root length

The results showed that there was significant difference between dilaceration and type of the teeth (*p*< 0.001). In comparison to other teeth, the highest prevalence of dilaceration was observed in maxillary canine (*p*= 0.006) (Figure 4). There was no significant relationship between the prevalence of dilaceration in anterior and canine teeth and gender (*p*= 0.670) (Table 1). The results obtained from this study showed that there was no significant difference between the prevalence of dilaceration and the type of jaw (*p*= 0.231) (Table 2). In general, the highest prevalence of dilaceration was in the apical third and towards the distal and buccal parts (*p*< 0.001). In terms of severity, the mild form was more common than the others were (*p*< 0.001) (Figure 5). Cohen’s kappa coefficients for the first and second evaluations were 0.991 and 0.993, respectively, with respect to the inter-examiner’s agreement (after the training session). The overall Cohen’s kappa coefficient for the intra-examiner’s agreement was 0.996. Altogether, this result demonstrates a very good agreement between the intra- and inter-examiners.

**Table 1:** The distribution of dilaceration between the two genders

	Female	Male	Total	*p*Value
Teeth without dilaceration	753 (93.7%)	692 (94.3%)	1445 (94.02%)	*p*= 0.670
Teeth with dilaceration	50 (6.3%)	42 (5.7%)	92 (5.98%)	
Total	803 (100%)	734 (100%)	1537 (100%)	

**Table 2:** The prevalence and distribution of the dilacerated teeth based on the jaws

	Maxilla	Mandible	Total	*p*Value
Teeth without dilaceration	743 (93.8%)	702 (94.2%)	1445 (94.02%)	*p*= 0.231
Teeth with dilaceration	49 (6.2%)	43 (5.8%)	92 (5.98%)	
Total	792 (100%)	745 (100%)	1537 (100%)	

## Discussion

The success of endodontic therapy depends on thorough understanding of tooth and root canal morphology and anomaly in different population and race [ 1 , 19 ]. One of the developmental anomalies is dilaceration, which can affect the success of root canal treatment [ 23 ]. It is important that with modern diagnostic tools, we are able to identify anomalies that change and improve treatment plan [ 1 ]. 

Some researchers defined a curvature of 90 degrees or more (relative to the longitudinal axis of the tooth) as dilaceration [ 4 - 5 ], while others have considered a curvature of 20 degrees or more as dilaceration in their studies [ 6 ]. The presence of dilaceration in high-resolution CBCT images was based on Chohayeb definition [ 6 ] and the dilaceration classification was based on Santana’s study [ 7 ].

Although many studies [ 2 , 4 , 14 ] have been conducted to assess the prevalence of dilaceration, their methodologies were different. Some studies used conventional periapical radiography [ 14 , 24 ], while others employed panoramic radiography along with periapical radiography [ 25 , 26 ]. In addition, some previous studies used extracted teeth [ 6 , 27 ].

The studies, which were conducted on extracted teeth, underestimated the amount of root curvature. The reason was that practitioners had great difficulty in extracting curved teeth with common methods and turned to surgery and sectioned their roots. 

Conventional radiographs usually provide information about the morphology of the roots in two dimensions and do not provide accurate information about the third dimension and the exact location of dilaceration on roots [ 14 , 28 ].

New studies on the anatomy of teeth have demonstrated that the anatomical malformations can be better detected by new advanced technologies such as CBCT along with conventional radiography. CBCT radiography has been widely used in studies on root morphology. It shows the structural nature of the tooth in three dimensions and determines the angles of root curvature with great precision [ 19 , 23 , 29 - 30 ]. The present study is one of the first studies in Iran to investigate the prevalence of dilaceration in the anterior and canine teeth of both jaws using CBCT images.

In our study, the prevalence of dilaceration in individuals with at least one tooth with this anomaly was 14.5%. Furthermore, the prevalence of dilaceration in all anterior and canine teeth was 5.98%. The maxillary canine had the highest amount of dilaceration which was statistically significant (*p*= 0.006). In a study by Kuzekanani *et al*. [ 31 ], the prevalence of anterior tooth dilaceration in the Iranian population was reported to be close to zero. Hamasha *et al*. [ 4 ] conducted a similar study on the Jordanian population and stated that the rate of this anomaly in the anterior teeth was about 1%. Moreover, in Nabavizadeh *et al*.’s study [ 14 ], the prevalence of dilaceration in anterior teeth was much lower than that of the present study. The differences between these results can probably be attributed to different study methods (using conventional radiography in other studies), dissimilar sample sizes, and different definitions of dilaceration considered in their studies (angle of greater than 90 degrees for the mesial and distal directions and the bull’s-eye view in the buccal and lingual directions); the latter is the most important one.

Based on the results in the present study and the study of Asheghi *et al*. [ 28 ] (first part of the present study), the prevalence of the studied anomaly was much lower in the anterior teeth than in the posterior teeth, which was in accordance to the study of Nabavizadeh *et al*. [ 14 ]. Similar results were found in the study of Ostovarrad *et al*. [ 32 ] who reported a prevalence of 2.8% and 11.1% in anterior and posterior teeth, respectively. These results were statistically significant and could also be useful in the relationship between trauma and dilaceration. The hypothesis that trauma could be the cause of dilaceration can be reevaluated because the prevalence of dilaceration was higher in posterior teeth. In fact, as confirmed by the findings of Miloglu *et al*. [ 2 ], its occurrence might be independent of trauma. On the other hand, ectopic root development and lack of space are probably the causes of the high prevalence of this anomaly in the third molars [ 5 ].

Ledesma-Montes *et al*. [ 33 ] found the highest prevalence of dilaceration in the second premolar and maxillary lateral teeth in contrast to our study. They concluded that the reason for this root curvature in the teeth was the lack of space for the eruption of teeth and the replacement of primary teeth by the associated permanent teeth [ 33 ].

The direction of the root curvature of lateral incisors was distal in most studies. Moreover, in Benitha *et al*.’s study [ 25 ], lateral incisors had the highest prevalence of dilaceration among the permanent incisors. Most of these dilacerations were towards the distal direction similar to our study.

In terms of the location of this anomaly in the anterior and canine teeth in our study, the most frequent location was the apical third and the least frequent location was the coronal part which is in line with the results of other studies [ 25 , 28 ]. Furthermore, in the present study, the most frequent directions were distal, buccal, mesial, and lingual, respectively. Similarly, in the studies of Aminsobhani *et al*. [ 34 ] and Silva *et al*. [ 35 ], the highest prevalence of this anomaly was reported in the distal direction.

Consistent with the results of other studies [ 2 , 26 , 36 ], the current study showed that the prevalence of dilaceration is independent of gender. Furthermore, in some studies, the results were contradictory. Some of them showed more prevalence of dilaceration in female [ 33 , 37 ], whereas the others reported that it was more in male [ 35 , 38 ].

In the present study, there was no significant difference in the maxillary and mandibular teeth regarding the prevalence of dilaceration, which was similar to the results of previous studies [ 2 , 14 , 33 ]. In the studies of Miloglu *et al*. [ 2 ] and Karadas *et al*. [ 26 ], the highest prevalence in anterior teeth belonged to maxillary lateral teeth. In Ostovarrad *et al*.’s study [ 32 ], the highest prevalence among anterior teeth was related to the maxillary teeth. Probably the reason for this difference is due to the different definition of the dilaceration, study method, and the studied population. 

Out of 1537 teeth, 92 teeth had dilaceration; most of which were in mild form (92.4%) and no severe dilaceration was found in any tooth. Furthermore, in the study conducted by Ostovarrad *et al*. [ 32 ] who used the same method of measuring the severity of dilaceration, the mild form of dilaceration had a higher prevalence (62. 9%). The results of other studies agreed with the findings of the current study as well as Estrela's study [ 39 - 40 ].

The prevalence of dilaceration in the anterior and canine teeth was shown to be notable in the current study. Since the dilaceration prevalence can be affected by the race of the study population, it would be recommended to investigate the prevalence of dilaceration in different population in the northern part of the country using CBCT.

## Conclusion

It is recommended that practitioners examine the canine teeth in both genders more carefully during root canal treatments. CBCT can be a complementary method for diagnosing dilaceration in these teeth because after the distal direction, the buccal direction was the most common dilaceration direction in this study, which cannot be easily detected on conventional radiography.

## Acknowledgements

The authors of this paper would like to appreciate the Vice-chancellery of Shiraz University of Medical Science for supporting this study (Ethics Committee Approval: #IR.SUMS.REC.1397.201)

## Conflict of Interests

The authors declare that they have no conflicts of interests.
